# Platelet surface receptor glycoprotein VI-dimer is overexpressed in stroke: The Glycoprotein VI in Stroke (GYPSIE) study results

**DOI:** 10.1371/journal.pone.0262695

**Published:** 2022-01-18

**Authors:** Isuru Induruwa, Harriet McKinney, Carly Kempster, Patrick Thomas, Joana Batista, Jean-Daniel Malcor, Arkadiusz Bonna, Joanne McGee, Elaine Bumanlag-Amis, Karola Rehnstrom, Sophie Ashford, Kenji Soejima, Willem Ouwehand, Richard Farndale, Kate Downes, Elizabeth Warburton, Masaaki Moroi, Stephanie Jung

**Affiliations:** 1 Department of Clinical Neurosciences, University of Cambridge, Cambridge, United Kingdom; 2 Department of Haematology, University of Cambridge, Cambridge, United Kingdom; 3 Department of Biochemistry, University of Cambridge, Cambridge, United Kingdom; 4 Research and Development Coordination and Administration Department, KM Biologics Co., Ltd, Kumamoto, Japan; Fiji National University, FIJI

## Abstract

**Objectives:**

Platelet activation underpins thrombus formation in ischemic stroke. The active, dimeric form of platelet receptor glycoprotein (GP) VI plays key roles by binding platelet ligands collagen and fibrin, leading to platelet activation. We investigated whether patients presenting with stroke expressed more GPVI on their platelet surface and had more active circulating platelets as measured by platelet P-selectin exposure.

**Methods:**

129 ischemic or hemorrhagic stroke patients were recruited within 8h of symptom onset. Whole blood was analyzed for platelet-surface expression of total GPVI, GPVI-dimer, and P-selectin by flow cytometry at admission and day-90 post-stroke. Results were compared against a healthy control population (n = 301).

**Results:**

The platelets of stroke patients expressed significantly higher total GPVI and GPVI-dimer (*P*<0.0001) as well as demonstrating higher resting P-selectin exposure (*P*<0.0001), a measure of platelet activity, compared to the control group, suggesting increased circulating platelet activation. GPVI-dimer expression was strongly correlated circulating platelet activation [r^2^ = 0.88, *P*<0.0001] in stroke patients. Furthermore, higher platelet surface GPVI expression was associated with increased stroke severity at admission. At day-90 post-stroke, GPVI-dimer expression and was further raised compared to the level at admission (*P*<0.0001) despite anti-thrombotic therapy. All ischemic stroke subtypes and hemorrhagic strokes expressed significantly higher GPVI-dimer compared to controls (*P*<0.0001).

**Conclusions:**

Stroke patients express more GPVI-dimer on their platelet surface at presentation, lasting at least until day-90 post-stroke. Small molecule GPVI-dimer inhibitors are currently in development and the results of this study validate that GPVI-dimer as an anti-thrombotic target in ischemic stroke.

## Introduction

Platelet activation and subsequent thromboembolism underpins the pathophysiology of ischemic stroke. Current antithrombotic agents used in stroke are effective at reducing ischemic events [1], but have serious systemic side effects such as bleeding [2]. There is a need, therefore, for more specific antithrombotic agents with a safer pharmacological profile.

In the last three decades, the platelet surface glycoproteins that regulate hemostasis and thrombosis have been extensively researched. Glycoprotein (GP) VI, which recognizes subendothelial collagen exposed at sites of atherosclerotic plaque rupture and initiates thrombus formation, has attracted much attention as a potential antithrombotic target [3]. The surface-expressed form of GPVI is a complex between a single GPVI molecule (GPVI monomer) and the FcR γ-chain dimer, which further dimerizes to generate the functional form of the receptor, here termed GPVI-dimer [4]. GPVI-dimers are constitutively present on the surface of circulating (resting) platelets, comprising 20–29% of total GPVI [5]. Platelet activation increases surface GPVI-dimer expression and binding to collagen induces GPVI-dimer clustering, which increases GPVI avidity, causing further platelet activation [6, 7]. Recently, GPVI has been shown to bind to fibrin and cause platelet activation [8, 9]. Our work has shown that GPVI-dimer recognizes and binds to the D-domain of fibrin(ogen) [10] and that GPVI-dimer binds with classical receptor kinetics to fibrin clots formed in vitro [11].

These GPVI interactions with collagen and fibrin make it a tantalizing target in ischemic stroke. The ability to activate platelets through adhering to collagen exposed during arterial plaque rupture initially, and later to stabilize a growing thrombus through the GPVI–fibrin interaction, suggest a key role in the pathophysiology of large artery ischemic stroke (LAS). Additionally, in cardioembolic stroke (CES), where systemic inflammation and endothelial dysfunction leads to platelet activation through the generation, first, of thrombin and then of fibrin [12, 13], GPVI could bind and support intracardiac thrombosis through its interaction with fibrin. Antibody-mediated GPVI-dimer inhibition has so far demonstrated positive results in early clinical trials [14], which suggests GPVI-dimer could be a future anti-thrombotic target in ischemic stroke.

To resolve the levels of platelet GPVI-dimer and total GPVI (monomeric and dimeric GPVI on the platelet surface) in patients with stroke, we used the GPVI-dimer–specific antibody 204–11 Fab [5] and HY101 [15], an antibody that recognizes both GPVI-monomers and -dimers. To quantify platelet activation, flow cytometry was used to measure the surface expression of P-selectin (a marker of platelet activation; also known as CD62P) in resting platelets and after stimulation by adenosine diphosphate (ADP), which activates platelets through purinergic receptors P2Y1 and P2Y12 [16], or by the GPVI-specific collagen-mimetic crosslinked Collagen-Related peptide (CRP-XL) [17].

The aim of the present study was to investigate whether patients presenting with stroke have inherently more active platelets and whether they express constitutively higher levels of GPVI-dimers, representing a potential controllable point of thrombosis in different etiologies of ischemic stroke.

## Methods

### Patient recruitment

The study was carried out between 2015 and 2018 at a teaching hospital and hyper-acute stroke unit in Cambridge, UK, which admits approximately 800 stroke patients annually. Patients with suspected ischemic or hemorrhagic stroke presenting at the emergency department (ED) within 8 hours of symptom onset were invited to participate in the study. For those unable to provide written informed consent at the time, their designated next of kin was approached, and the patient re-consented if capacity was regained. Exclusion criteria were age <18 years, pregnancy, known past or active malignancy, platelet disorder or abnormal platelet count (<150 x 10^9^/L), low hemoglobin (Hb: <9.5 g/L), transient ischemic attack (TIA), myocardial infarction, HIV, or hepatitis. Patients subsequently diagnosed with an alternative diagnosis to stroke were also excluded. All patients with confirmed ischemic stroke were invited to give a repeat blood sample as close as possible to day 90 post-stroke. The study protocol was approved by the East of England Essex Ethics Committee (ref 14/EE/1062).

### Control population recruitment

Healthy donors without previous thrombotic disease (ischemic heart disease, myocardial infarction or stroke), known platelet disorders or malignancy from the National Institute for Health Research Cambridge BioResource were invited to participate in the study. These healthy donors attended an appointment for blood sampling for the study, where written consent was obtained for participation. The study protocol was given ethics approval by the Cambridge East Ethics Committee (ref 10/H0304/65).

### Blood collection

Control and patient blood samples were drawn from the antecubital fossa vein using a 21-gauge butterfly needle. The first tube drawn was a K2 EDTA Vacutainer^®^ (BD, Franklin Lakes, NJ), which was solely used for full blood count quantification. Next, blood was drawn into a 0.109 mol/L sodium citrate Vacuette^®^ (Greiner, Kremsmünster, Austria). To avoid artefactual platelet activation, after removing the tourniquet, the first 5 mL was discarded, and blood was then taken to the laboratory on site for immediate analysis. A Sysmex XN-1000 (Sysmex, Kobe, Japan) was used to carry out a full blood count of all control and participant samples at the same time as GPVI and platelet function measurements. For ischemic stroke patients only, a second blood sample was obtained and analyzed using the same methods at 90 days later. when they attended their routine follow-up appointment.

### Quantification of total GPVI (monomer and dimer) and GPVI-dimer levels by flow cytometry

Citrated whole blood was diluted 5-fold with HEPES-Buffered Saline (HBS: 0.15 M NaCl, 5 mM KCl, 1 mM MgSO_4_, 10 mM HEPES, pH 7.4; Sigma-Aldrich Ltd, Gillingham, UK). The relevant primary antibody was added: either anti-total GPVI HY101, 12.5 μg/mL (IBGRL Bristol, UK); anti-GPVI-dimer 204–11 Fab, 5 μg/mL (developed by MM and SMJ, in collaboration with Kaketsu-ken, Kumamoto, Japan); IgG2a negative control, 12.5 μg/mL (BioLegend, London, UK); or mouse Fab negative dimer control, 5 μg/mL (BioLegend) and incubated at room temperature for 10 min. The fluorescent secondary antibody FITC-F(ab’)_2_ (Jackson Laboratory, ME) was added at 40 μg/mL and incubated in the dark for 10 min. The sample was subsequently diluted in 0.5 mL HBS prior to measurements using the FC500 flow cytometer (Beckman-Coulter Ltd., High Wycombe, UK). Platelets were identified using light scatter and results were recorded as mean fluorescence intensity (MFI).

### Platelet activation measurements using whole blood flow cytometry

Platelet activation was measured by detecting platelet surface expression of the activation marker P-selectin, as previously described [18]. A 5 μL aliquot of aspirinated (100 μM) and hirudinized (4 U/mL) citrated whole blood was added to HBS after 5-min incubation, to make up a final volume of 50 μL containing PE-conjugated anti-CD62P (1:50 dilution) (Clone Thromb6; Bristol Institute for Transfusion Science, Bristol, UK) and either no agonist; 0.5 μM ADP, final concentration (Sigma-Aldrich); or 4 μg/mL CRP-XL, final concentration (Farndale Laboratory, University of Cambridge [Now available from CambCol Laboratories, Ely, UK]). For the CRP-XL–stimulated platelets, apyrase (4 U/mL, final concentration, Sigma-Aldrich) was added to inhibit activation through the ADP-induced pathway before adding CRP-XL.

Activation reactions were stopped after 20 min by adding 100-fold volume of saline containing 0.2% formyl solution (37% formaldehyde, 0.85% NaCl; Sigma-Aldrich) and after a 10-min incubation, flow cytometry was carried out. Negative controls for the anti-P-selectin were set using a 9E10 isotype control (Bristol Institute for Transfusion Science, Bristol, UK). Platelets were identified by light scatter and results were recorded as the percentage of platelets positive (%PP) for P-selectin, which was calculated as the percentage of platelets expressing P-selectin with MFI greater than 98% of the isotype control.

### Clinical data collection

For the control and stroke populations, demographic variables including age, sex and history of relevant clinical risk factors [hypertension, congestive cardiac failure (CCF), diabetes mellitus, hypercholesterolemia, ischemic heart disease (IHD), previous stroke and atrial fibrillation (AF)] were collected. Anti-hypertensive, statin, antiplatelet and anticoagulation prescriptions were documented for both cohorts. In addition, for the stroke group, admission parameters including national early warning score (NEWS) and blood C-reactive protein (C-RP) at admission were recorded. Stroke-specific scores including National Institutes of Health Stroke Scale (NIHSS), Modified Rankin Score (mRS) at discharge as well as a pre-stroke CHA_2_DS_2_-VASc score were collected. CHA_2_DS_2_-VASc scores were calculated for all participants, not just patients with AF, as it gives a composite score of stroke risk factors [19]. Electronic discharge letters from the direct care teams and inpatient medical records, which contain admission information and stroke etiology, were used to subclassify ischemic strokes as per the TOAST classification [20]: LAS, CES, small vessel occlusion (SVO), stroke of other aetiology (Other), stroke of undetermined aetiology (UD). Hemorrhagic strokes were not sub-classified. Electronic records were re-checked 6 months from discharge to see if stroke etiology was deemed to have changed from the original classification.

### Statistical analysis

A power analysis was performed for sample size estimation based on pilot data from previous GPVI-dimer quantification. The effect size was 10% and sample size calculations for a 2-sided test with a Type I error of 0.05 and power of 0.80 determined that <20 participants were required in each group. A *P-*value of <0.05 was taken as statistically significant. Linear regression revealed that age and mean platelet volume (MPV) were significantly associated with GPVI expression in the stroke patients. Therefore, GPVI expression and P-selectin exposure results from control and stroke groups were adjusted for age and MPV and predicted values calculated from adjusted unstandardized residuals were subsequently used when comparing mean values between cohorts. Specific statistical tests employed are described in the figure legends.

Age- and MPV-adjusted values were also entered into a simple linear regression analysis to determine association between total GPVI or GPVI-dimer expression with other single predictor variables. Significantly associated predictor variables (*P*<0.05) were then entered into a multiple regression model to determine independent predictors for GPVI-dimer expression. Unstandardized coefficient (B), standard error (SE) and significance (*P*) are reported for each of the significantly associated variables. Data was analyzed using Prism v9.0 (GraphPad, San Diego, CA) and SPSS v.27 (IBM Corp., Armonk, NY).

## Results

### Recruitment and population details

Results from 301 healthy and contemporaneous controls were included in the study (Table 1). A total of 247 patients were also recruited, totalling 186 stroke patients and 61 non-stroke patients (who presented with stroke-like symptoms but were subsequently found to have an alternative diagnosis and were then excluded from analysis). 57 stroke patients were also excluded due to meeting exclusion criteria; presence of malignancy, blood/platelet disorder, transient ischaemic attack (TIA) as well as incomplete blood collection, errors in sample analysis or donor request. Therefore, the final set of data includes GPVI and platelet function results from 129 stroke patients. The mean (±SD) time between venepuncture and processing for the controls was 16±4 minutes. For the stroke patients the mean time between arrival to ED and venepuncture was 129±21 minutes and the time between venepuncture and processing was 34±8 minutes.

**Table 1:** Baseline characteristics of control and stroke groups at admission, including haematological parameters, vascular risk factors and admission details.

	Control	Stroke day-0	*P*
N	301	129	
Age (Years)	60 (47–68)	79 (67–85)	**<0.0001**
Female (%)	65.8	38.0	**<0.0001**
Hemoglobin (g/L) ± SD	136.4±11.4	139.8±17.9	**0.02**
Platelet Count (10^9^/L)± SD	260.2±57.3	239.6±88.1	**0.004**
Mean platelet volume (fL)±SD	10.4±0.9	10.80±0.99	**0.007**
CHA_2_DS_2_-VASc Score	1 (1–2)	3 (2–4)	**<0.0001**
Admission NIHSS	NA	8 (4–15)	NA
Admission C-RP	NA	3.38 (1–7)	NA
Admission NEWS	NA	1 (0–2)	NA
Thrombolyzed, n (%)	NA	50 (38.8)	NA
Thrombectomy, n (%)	NA	6 (4.7)	NA
***Risk factors for thrombotic disease*, *n (%)***			
Atrial Fibrillation	2 (0.66)	61 (47.3)	**<0.0001**
Congestive Cardiac Failure	0 (0)	9 (7.0)	**<0.0001**
Hypertension	27 (9.0)	94 (72.9)	**<0.0001**
Diabetes	1 (0.33)	24 (18.6)	**<0.0001**
Ischemic Heart Disease	0 (0)	24 (18.6)	**<0.0001**
Cholesterol	24 (8.0)	50 (38.8)	**<0.0001**
Previous stroke	0 (0)	26 (20.2)	**<0.0001**
***Admission medication*, *n (%)***			
ACE inhibitor or ARB	21 (7.0)	24 (18.6)	**0.0006**
Aspirin	1 (0.33)	33 (25.6)	**<0.0001**
Clopidogrel	0 (0)	7 (5.4)	**0.0003**
Apixaban	0 (0)	1 (0.8)	0.66
Dabigatran	0 (0)	1 (0.8)	0.66
Rivaroxaban	1 (0.33)	3 (2.3)	0.44
Warfarin	0 (0)	9 (7.0)	**<0.0001**
Statin	22 (7.3)	36 (27.9)	**<0.0001**

Means are reported with standard deviation and medians, with interquartile range in parentheses. Significance of differences between cohorts were analysed by an unpaired t-test for parametric data and Chi-squared test used for non-parametric data and are reported as a *P-*value. SD = standard deviation, IQR = interquartile range, CHA_2_DS_2_-VASc (assigns 1 point for history of congestive cardiac failure (C), hypertension (H), diabetes mellitus (D), vascular disease (V), age ≥ 65 years (A) and female sex (Sc), and 2 points if age ≥75 years (A_2_) or there is a history of prior stroke/transient ischaemic attack (S_2_), NIHSS = National Institutes of Health Stroke Scale, C-RP = C-reactive protein, NEWS = National Early warning Score, ACE = angiotensin-converting-enzyme, ARB = angiotensin-receptor blocker. NA = not applicable.

Results from 51 ischemic stroke patients who attended follow-up [median days (IQR): 110 (92–128)] were available and will be referred to as ‘day-90’ herein. No patients were excluded from the study on the basis of stroke severity or acuity of illness. Demographic details, blood parameters, comorbidities, admission scores and medication of the control, and stroke populations are included in Table 1.

The age between the control and stroke populations were significantly different [median years (IQR): controls, 60 (47–68); stroke, 79 (67–85), *P* <0.0001] as well as MPV (mean±SD: controls 10.4±0.9; stroke 10.80±0.99, *P* = 0.007). Therefore, raw data for GPVI expression and platelet function were adjusted by both age and MPV in the control and stroke groups and these adjusted values were used in any subsequent analysis, as described in the statistical analysis section. After age and MPV adjustment, the control GPVI expression and P-selectin exposure results fell within a narrower range of values compared to the stroke population. This is to be expected as the stroke patients would have variation in their GPVI expression and platelet function beyond that explained by age and MPV (e.g., caused by other vascular risk factors). Further analysis revealed that gender was not related to total GPVI (*P* = 0.30), GPVI-dimer (*P* = 0.91) or resting P-selectin exposure (*P* = 0.96).

The control population had fewer stroke risk factors compared to the stroke group (*P* <0.0001) and as expected, a higher proportion of stroke patients were taking aspirin, clopidogrel and a statin at admission compared to the control group (*P* <0.0001).

At admission, the stroke group had a median NIHSS of 8 (IQR 4–15). 50 patients received thrombolysis with alteplase and 6 underwent thrombectomy. 33/50 patients who were thrombolyzed had their blood taken after receiving thrombolysis. There were no differences between total GPVI or GPVI-dimer expression or resting P-selectin exposure between patients who were thrombolyzed compared to those who were not or if blood sampling was performed pre- or post-thrombolysis (S1 Fig).

### Stroke patients express more GPVI and P-selectin on their platelet surface

**Fig 1:**
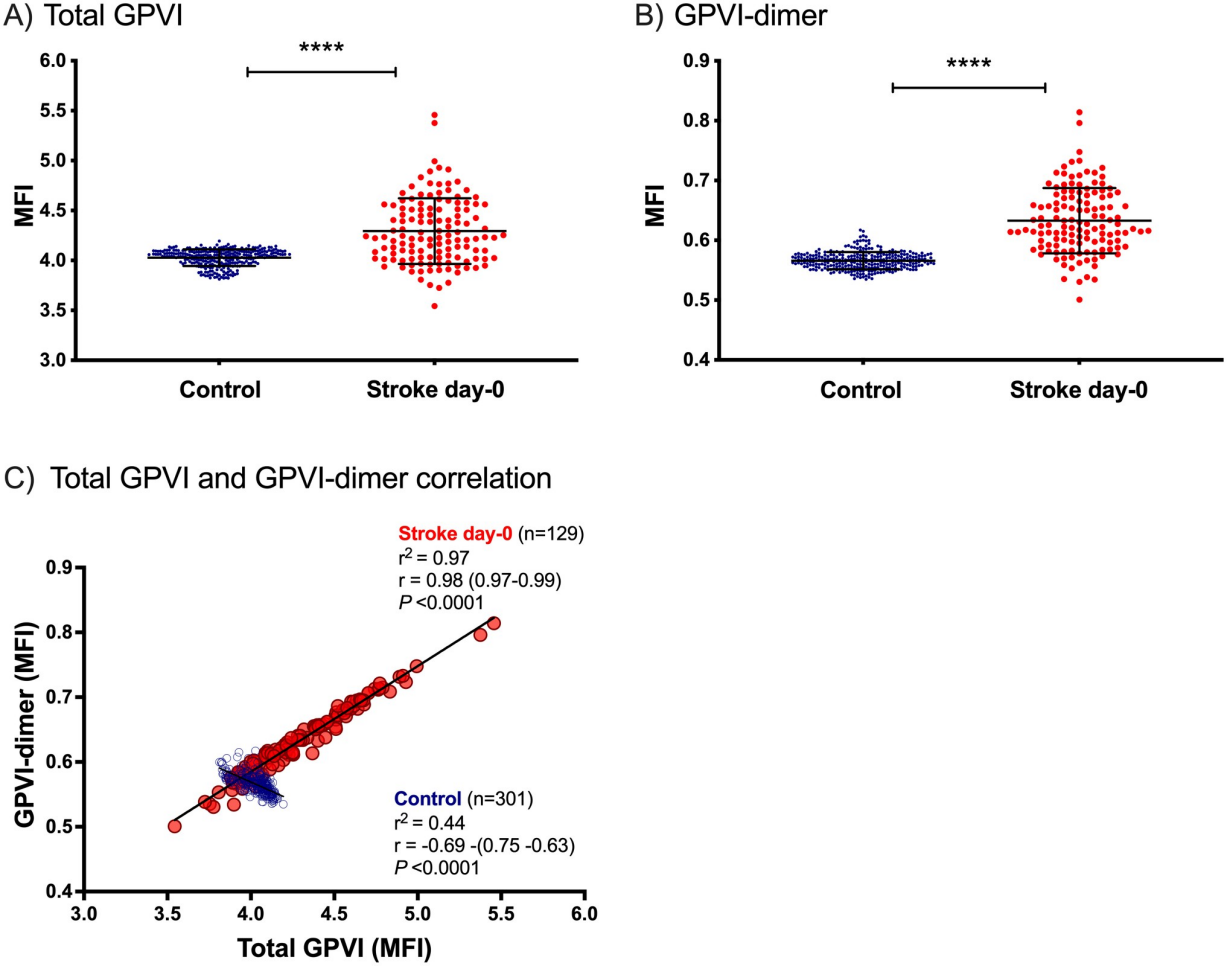
Comparison of GPVI expression between control and stroke cohorts and GPVI-dimer as a function of total GPVI in control and stroke cohorts. A) Total GPVI and B) GPVI-dimer are both significantly higher in the stroke population at admission compared to the control cohort (*P* <0.0001). *P*-values were calculated using an unpaired t-test and the error bars represent the mean MFI of each of the cohorts ± SD. C) Correlation between platelet surface total GPVI-dimer and GPVI-dimer. These data were calculated using Pearson correlation coefficient and the regression line is presented for each set of data. The control platelets show a narrow range of GPVI-dimer levels, which is negatively correlated to total GPVI (*P* <0.0001). In contrast, the surface GPVI-dimer of stroke patients’ platelets demonstrate strong positive correlation to the amount of total GPVI (*P* <0.0001) and in general have higher GPVI-dimer levels than the controls. MFI = mean fluorescence intensity.

Compared to controls, the stroke patients expressed significantly higher total GPVI (MFI±SD: control, 4.03±0.08; stroke, 4.30±0.32; P <0.0001) (Fig 1A) and GPVI-dimer (MFI±SD: control, 0.57±0.01; stroke, 0.63±0.05; P <0.0001) (Fig 1B). Control GPVI-dimer expression was negatively correlated with total GPVI. In contrast, platelet surface GPVI-dimer expression was strongly correlated with total GPVI in stroke patients (r^**2**^ = 0.97, P <0.0001; Fig 1C).

**Fig 2:**
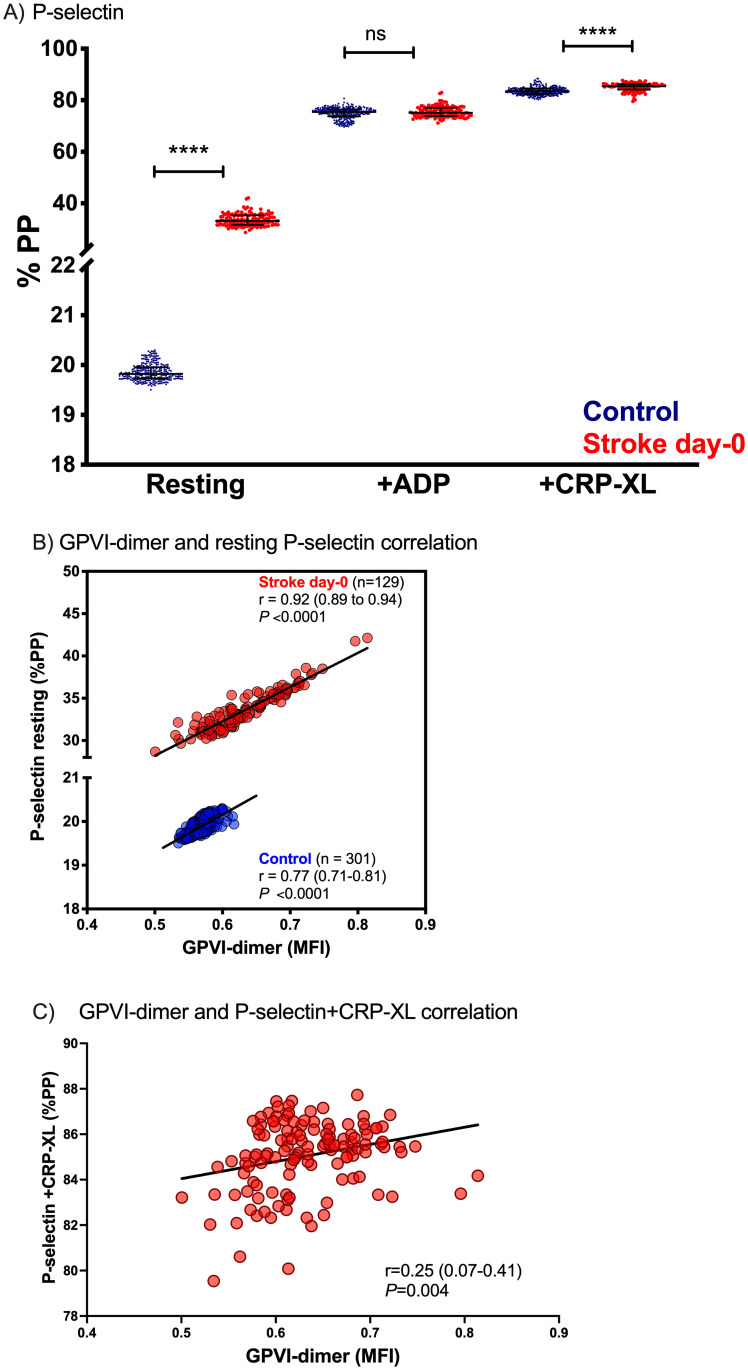
Comparison of P-selectin exposure between control and stroke cohorts and correlation between GPVI-dimer and P-selectin exposure. A) Comparison of P-selectin surface exposure of the stroke patients with the controls show that the stroke patients have more activated “resting” platelets (i.e., before stimulation by exogenous agonist) and after CRP-XL (4 μg/mL) addition compared to the control group (*P*<0.0001). P-selectin exposure in response to ADP (0.5 μM) was not significantly different compared to controls. The error bars represent the median (Q1–Q3) %PP of each of the cohorts and *P*-values were calculated by the Mann-Whitney U-test for non-parametric data. B) GPVI-dimer expression and resting P-selectin exposure in both control and stroke patients are strongly correlated at admission (*P* <0.0001 for both). C) GPVI-dimer expression is correlated with P-selectin exposure in response to CRP-XL in the stroke patients at admission (*P* = 0.004). Correlations were calculated using Spearman’s rank correlation coefficient and regression line is presented for each data set. %PP = percentage of platelets positive, ns = not significant.

Resting P-selectin exposure, a platelet activation marker, was significantly higher in the stroke patients (Fig 2A), compared to controls (P <0.0001). Fig 2B shows P-selectin expression in resting platelets (i.e., platelets with no added agonist) as a function of surface GPVI-dimer. The resting control platelets show a low level of P-selectin that was correlated with the amount of GPVI-dimer in the control population (r = 0.77, P <0.0001). By contrast, the resting platelets of stroke patients showed markedly higher P-selectin and a much stronger dependence on platelet surface GPVI dimer level (r = 0.92, P <0.0001). There was also a correlation between GPVI-dimer expression and P-selectin exposure after CRP-XL addition (r = 0.25, P = 0.004; Fig 2C). These results suggest that the stroke patients may have platelets that are already in an activated or primed state and this activation is at least partly dependent on their expression of GPVI-dimer.

### GPVI-dimer but not total GPVI is elevated at 90-days post-stroke

Out of the 129 stroke patients, 114 had had ischemic strokes. From those, excluding those who had died, discharged to nursing care, were too frail to attend follow-up or those originally from another geographical area, 51 ischemic stroke patients who attended their 3-month follow-up had consented to give a repeat blood sample where total GPVI, GPVI-dimer and platelet function were quantified as before. Those who were not followed-up were significantly older (*P* = 0.002), more disabled as per discharge mRS (*P* <0.0001) and a significantly higher proportion had died by six months (*P* = 0.0004).

**Fig 3:**
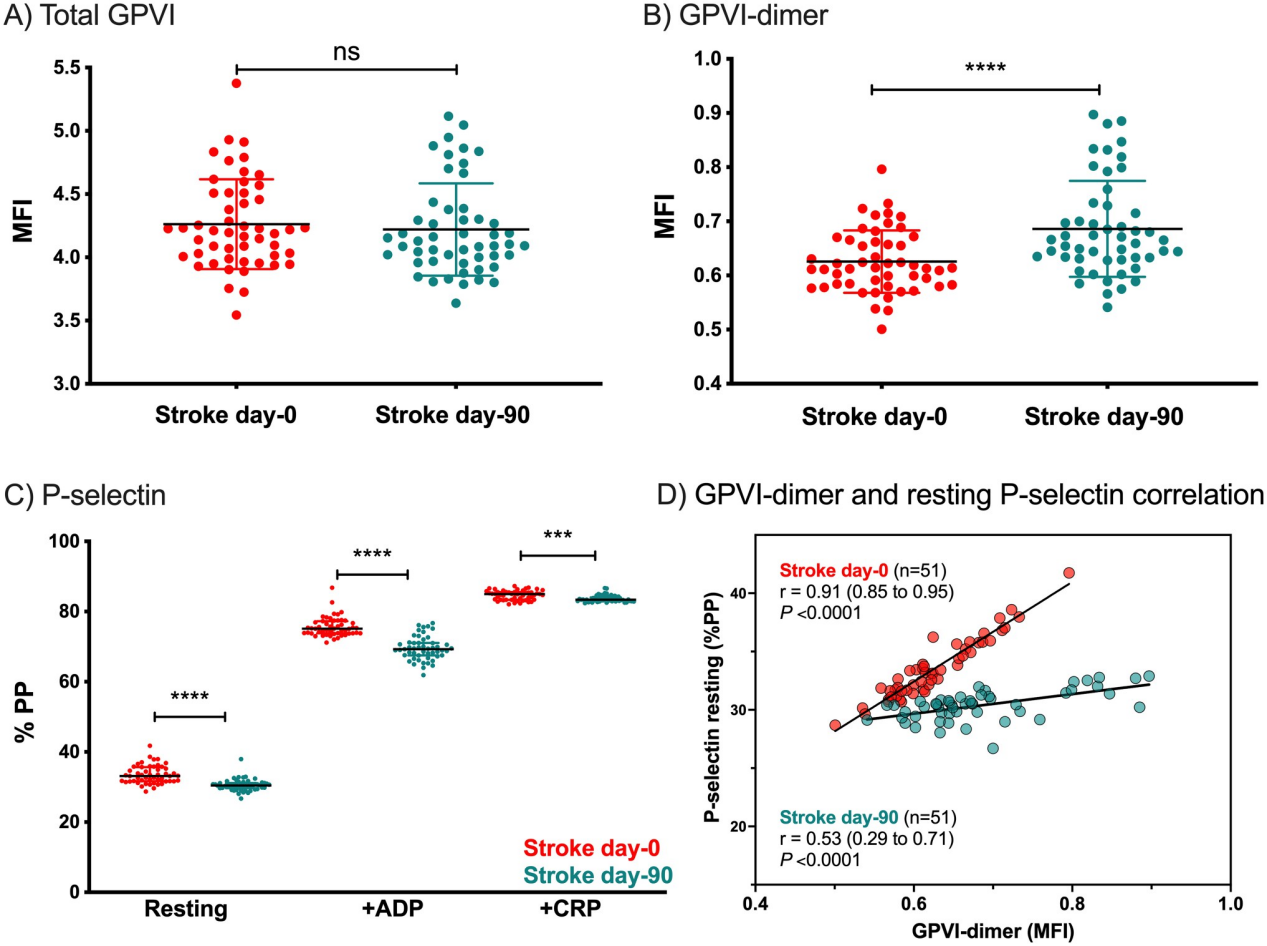
Comparison of GPVI expression and P-selectin exposure in stroke patients at day-0 and at day-90 (n = 51). A) Total GPVI expression is not different at day-90 compared to day-0 (*P* = 0.52). B) GPVI-dimer is significantly higher at day-90 compared to day-0 (*P* <0.0001). The error bars represent the mean MFI of each of the cohorts ± SD and *P*-values were calculated by a paired t-test. C) Resting P-selectin exposure (*P* <0.0001), P-selectin exposure induced by 0.5 μM ADP (*P* <0.0001), and P-selectin exposure induced by 4 μg/mL CRP-XL (*P* = 0.001) are all significantly lower at day-90. The error bars represent the median (Q1–Q3) %PP of each of the cohorts and *P*-values were calculated by the Wilcoxon signed-rank test for non-parametric data. ns = not significant. D) Comparison of P-selectin expressed on the platelet surface of stroke patients at day-0 and at day-90 after stroke and P-selectin of resting normal platelets. The P-selectin exposure on stroke patients at day-0 are strongly correlated to GPVI-dimer expression (*P <*0.0001) and higher than those of resting control platelets. By day-90, the stroke patients’ P-selectin exposure, albeit still correlated with GPVI-dimer level (*P* <0.0001), has decreased. Correlations were calculated using Spearman’s rank correlation coefficient and regression line is presented for each data set. MFI = mean fluorescent intensity, %PP = percentage of platelets positive.

Comparison of day-0 to day-90 platelets reveals that at follow-up, stroke patients at day-90 demonstrated no difference in total GPVI expression (day-0 MFI±SD: 4.26±0.36; day-90, 4.22±0.36; P = 0.52) (Fig 3A). However, GPVI-dimer expression was significantly higher at day-90 (day-0 MFI±SD: 0.63±0.05; day-90, 0.69±0.09; P<0.0001) (Fig 3B), even though their resting P-selectin expression was significantly lower (Fig 3C). Responsiveness to ADP or CRP-XL, measured as P-selectin exposure, were also significantly lower at day-90 (Fig 3C). In Fig 3D, the correlation between GPVI-dimer levels and P-selectin expression is compared for patient platelets at day-0 and day-90. Compared to the marked dependence of P-selectin exposure on GPVI-dimer levels in the day-0 stroke patients (r = 0.91, P <0.0001), their day-90 P-selectin exposure was only mildly dependent on surface GPVI-dimer (r = 0.53, P <0.0001).

Among the patients examined on both Day-0 and at Day-90 are 11 patients that had not been on clopidogrel initially but were newly started on clopidogrel by their day-90 appointment (S2 Fig). At day-90, they showed higher GPVI-dimer levels (*P* = 0.007, S2A Fig), but no differences in total GPVI (*P* = 0.68, S2B Fig). Compared to day-0, they had lower P-selectin exposure in their resting platelets (*P* = 0.001, S2C Fig) and lower P-selectin after ADP stimulation (*P* = 0.002, S2D Fig, but P-selectin in response to CRP-XL was not different (*P* = 0.90, S2E Fig).

### Total GPVI, GPVI-dimer, and P-selectin expression in different stroke subtypes

All ischemic stroke patients were sub-classified by stroke etiology as described in the methods. Hemorrhagic stroke (bleed) was not further sub-classified and sub-arachnoid hemorrhages were not recruited. 129 patients were categorized into LAS (n = 17), CES (n = 56), SVO (n = 18), Other (n = 2), UD (n = 21) and bleed (n = 5). Data from stroke patients classified into strokes of other etiology were excluded due to low numbers and UD were excluded due to ambiguity in interpreting their etiological significance.

**Fig 4:**
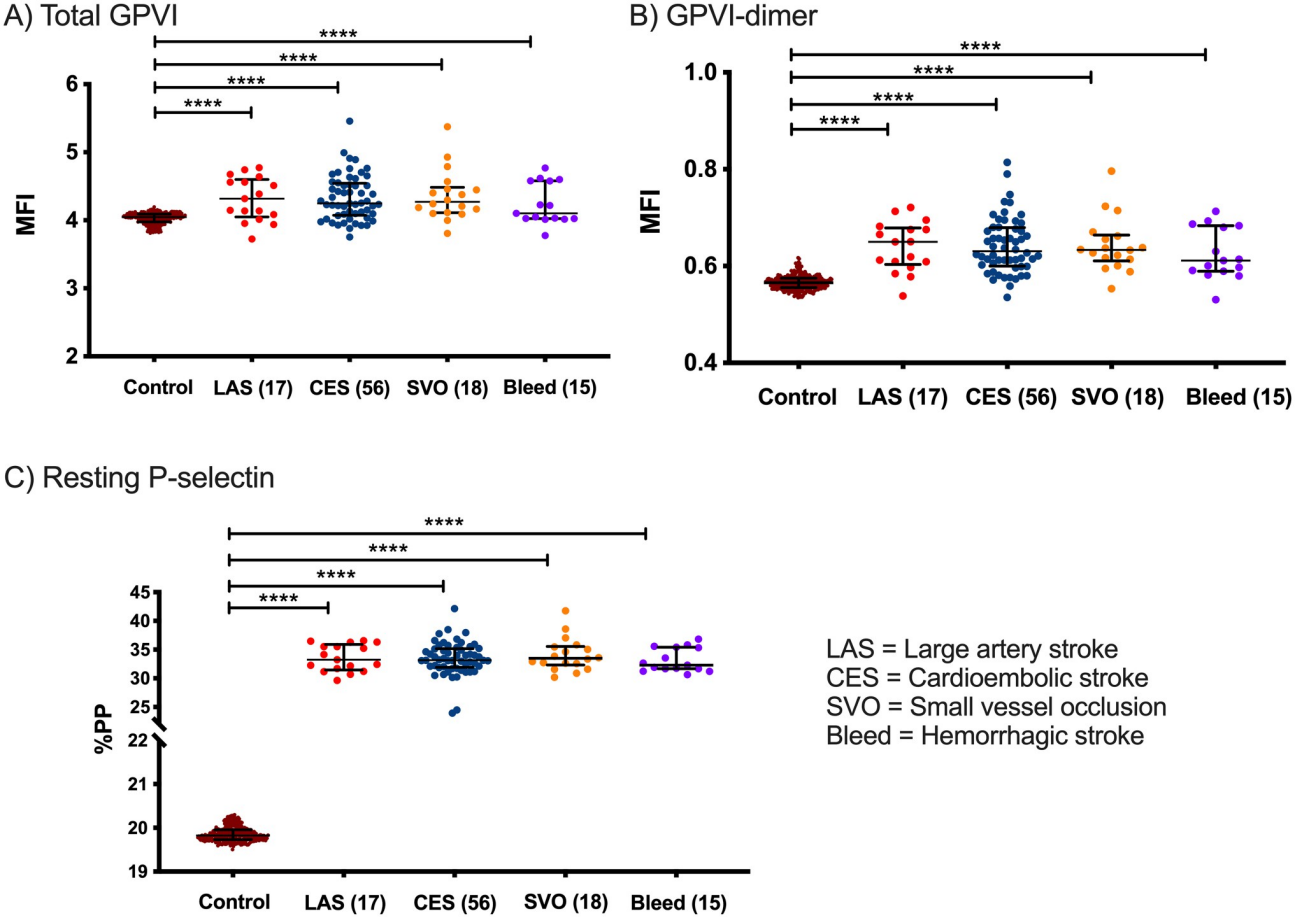
GPVI expression between control and stroke subtypes. A) Total GPVI expression (*P* <0.0001) and B) GPVI-dimer expression are higher in all ischemic stroke etiologies as well as hemorrhagic strokes (*P* <0.0001). *P*-values were calculated using one-way ANOVA with Dunnett’s multiple comparisons testing and the error bars represent the mean MFI of each of the cohorts ± SD. C) Resting P-selectin expression is higher in ischemic and hemorrhagic stroke (*P* <0.0001). *P*-values were calculated by a Kruskal-Wallis test with Dunn’s multiple comparisons testing for non-parametric data and the error bars represent the median (Q1–Q3) %PP of each of the cohorts. Numbers in parentheses show the number of participants within each group. MFI = mean fluorescent intensity, %PP = percentage of platelets positive.

All stroke subtypes (LAS, CES, SVO, bleed) demonstrated significantly higher total GPVI (Fig 4A, *P* <0.0001) and GPVI-dimer expression (Fig 4B, *P* <0.0001) as well as P-selectin exposure (Fig 4C, *P* <0.0001), compared to the controls, suggesting that patients with all stroke sub-types have more activated circulating platelets and higher GPVI expression.

### AF, hypertension and diabetes are independent predictors of GPVI levels in stroke population

Simple linear regression was used to determine the relationship between total GPVI or GPVI-dimer expression and a single predictor variable (Table 2). In stroke patients, the presence of AF and diabetes were both significantly associated with total GPVI (AF: B = 0.13, SE = 0.06, *P* = 0.02; diabetes: B = 0.2, SE = 0.07, *P* = 0.008) and GPVI-dimer (AF: B = 0.02, SE = 0.009, *P* = 0.01; diabetes: B = 0.03, SE = 0.01, *P* = 0.01) expression. Hypertension was also significantly associated with GPVI-dimer expression only (B = 0.02, SE = 0.01, *P* = 0.02).

**Table 2:** Simple linear regression to identify associations between total GPVI or GPVI-dimer expression and a single predictor variable in the stroke population.

	Total GPVI		GPVI-dimer	
	Coefficient B	Significance (P)	Coefficient B	Significance (P)
Female	0.08	0.07	0.02	0.08
**Comorbidities**				
**Atrial Fibrillation**	**0.13**	**0.02**	**0.02**	**0.01**
CCF	0.06	0.58	0.01	0.47
**Hypertension**	0.12	0.07	**0.02**	**0.02**
**Diabetes**	**0.20**	**0.008**	**0.03**	**0.01**
Ischemic Heart Disease	0.1	0.18	0.01	0.10
Cholesterol	0.01	0.82	0.002	0.87
Previous Stroke	–0.01	0.91	–0.003	0.81
**Admission and discharge parameters**				
Antiplatelets at admission	0.00	0.89	0.00	0.78
**CHA**_**2**_ **DS**_**2**_ **-VASc score**	**0.05**	**0.001**	**0.01**	**<0.0001**
**Admission NIHSS score**	**0.01**	**0.02**	**0.002**	**0.009**
Admission C-RP	0.00	0.83	0.00	0.65
Admission NEWS score	0.005	0.78	0.01	0.60
**Discharge mRS**	0.02	0.16	**0.005**	**0.03**
Death within six months	0.08	0.23	0.02	0.12

Significant associations to either total GPVI or GPVI-dimer expression (*P* <0.05) are presented in bold font. NIHSS = National Institutes of Health Stroke Scale, C-RP = C-reactive protein, NEWS = National Early Warning Score, mRS = Modified Rankin Score.

Furthermore, parameters such as the CHA_2_DS_2_-VASc score of all stroke patients and admission NIHSS and discharge mRS were both associated with total GPVI (CHA_2_DS_2_-VASc: B = 0.05, SE = 0.02, *P* = 0.001; NIHSS: B = 0.01, SE = 0.005, *P* = 0.02) and GPVI-dimer expression (CHA_2_DS_2_-VASc: B = 0.01, SE = 0.002, *P*<0.0001; NIHSS: B = 0.002, SE = 0.001, *P* = 0.009). Discharge mRS was also associated with GPVI-dimer expression (B = 0.005, SE = 0.002, *P* = 0.03), but not total GPVI (Table 2). Neither gender nor any of the markers of illness acuity measured on admission within the stroke population (C-RP and NEWS) were associated with GPVI expression. In multiple linear regression, the pre-admission CHA_2_DS_2_-VASc score was the strongest independent predictor of GPVI-dimer expression in stroke patients at admission (B = 0.008, SE = 0.004, *P* = 0.03) (S1 Table).

## Discussion

Previous studies investigating GPVI in thrombotic disease have focussed on either quantifying soluble GPVI (sGPVI), the metalloproteinase-cleaved ectodomain of GPVI shed from the platelet upon activation [21, 22], or on platelet surface expression of total GPVI (the sum of monomeric and dimeric GPVI) [23]. In these studies, stroke and TIA patients consistently demonstrated higher platelet expression of total GPVI [23]. However, an increase in total GPVI would not distinguish between an increase in GPVI-monomers, which comprise the majority of GPVI in circulating platelets, and the functional form, GPVI-dimer, an increase in which may be directly relevant to increased thrombogenicity. Moreover, it has been established that constitutive dimer levels in resting platelets increase upon platelet activation [5] and thus dimer expression would be an indicator of the activation state of the patients’ platelets. Keeping this in mind, if indeed ischemic stroke patients express more GPVI-dimer compared to healthy controls, GPVI-dimers might represent a point for pharmacological control of thrombus formation in ischemic stroke, especially as GPVI is a promising target in thrombus inhibition [3].

The novel findings of this study are that (1) platelet GPVI-dimer expression is significantly higher in stroke patients; (2) GPVI-dimer expression, but not P-selectin and total GPVI, is significantly higher in ischemic stroke patients at 90 days post-stroke compared to admission; (3) both ischemic and hemorrhagic sub-classes of stroke patients express significantly more GPVI-dimer; (4) the presence of AF, hypertension, diabetes and a higher CHA_**2**_DS_**2**_-VASc score, were associated with higher GPVI-dimer expression and (5) GPVI expression is associated with admission stroke severity as measured by NIHSS.

What is yet to be conclusively established is whether the over-expression of GPVI-dimer seen in stroke patients is due to the presence of vascular risk-factors, due to the acute stroke itself, or both. Evidently, the stroke cohort had a significantly higher burden of vascular risk factors (Table 1). Furthermore, a risk-factor driven element in GPVI-dimerization is evidenced from our results since AF, an inflammatory condition known to modulate platelet activity [24]; hypertension; diabetes; and a higher CHA_**2**_DS_**2**_-VASc score were all significantly associated with higher GPVI-dimer expression (Table 2). In previous work, Loyau et al. reported coronary artery disease patients had activated platelets showing GPVI-dimer expression, as measured by their dimer-specific antibody 9E18 [25]. More recently, Barrachina and colleagues demonstrated that both total GPVI and GPVI-dimer levels are elevated in platelets of obese patients without other comorbidities [26]. Both these studies, as well as this one, where the CHA_**2**_DS_**2**_-VASc score—a composite score of vascular risk factors—was the strongest independent predictor of GPVI-dimer expression (S1 Table) suggests that chronic low-level inflammation, driven by vascular risk-factors, may be a common characteristic among individuals who are also at higher risk of stroke. Therefore, it is likely that the stroke patients’ platelets expressed more GPVI-dimer, compared to controls, prior to their stroke.

Furthermore, we found a strong positive correlation between resting P-selectin exposure and GPVI-dimer expression in resting platelets of the stroke cohort (Fig 2B), similar to what had been demonstrated previously in coronary artery disease patients by Loyau et al. [25] In the controls, a positive relationship was also observed, although P-selectin expression was much lower. This is further evidence that platelet activity could be closely linked to GPVI expression prior to stroke and that platelets in the stroke group may circulate in a pre-activated or primed state. This is consistent with their significantly higher response to the GPVI-specific agonist CRP-XL compared to controls (Fig 2A) as well as the significant correlation between GPVI-dimer and P-selectin exposure with CRP-XL (Fig 2C).

At day-90 follow-up, although total GPVI was unchanged relative to day-0 (Fig 3A), GPVI-dimer expression was still significantly higher (Fig 3B). At this time, there is still a low but significant level of platelet activation and the P-selectin exposure is still linearly dependent on the patients’ GPVI-dimer level, although this is less dramatic than day-0 (Fig 3D, P≤ 0.0001, for both day-0 and day-90). Al-Tamimi et al. [21] reported that sGPVI was elevated in the plasma of ischemic stroke patients. To reconcile those observations with our own, we suggest that under the acute stroke conditions of day-0, patients’ platelets are highly activated and have marked increases in both total GPVI and GPVI-dimers, compared to control platelets. However, this increase in GPVI expression must also be in the setting of GPVI shedding from activated platelets during the thrombotic event, as reported by Al-Tamimi et al. Therefore, the apparent higher level of GPVI-dimer by day-90 may in fact more accurately reflect the constitutive number of dimers in the patients’ platelets prior to stroke and prior to any GPVI shedding during thrombosis. Given the 3-month life-span of platelets, increased GPVI-dimerization may also continue concomitantly with some shedding post stroke, driven by a pro-thrombotic propensity in the patients’ blood after stroke.

P-selectin exposure in resting, ADP-induced, and CRP-XL-induced platelets were all lower at day-90 (Fig 3C). This is consistent with previous work demonstrating an acute rise in P-selectin expression at presentation with stroke, with a gradual decrease in P-selectin thereafter [27, 28]. What is interesting, however, is that P-selectin exposure in response to both ADP and CRP-XL at day-90 is lower than that at admission, despite GPVI-dimer expression being higher. At least in part this is an effect of medication, as many patients would have been newly commenced on antiplatelet or anticoagulation therapy post-stroke. In the limited cohort of patients (11 individuals) who were not taking anti-thrombotics on or before day-0 but given clopidogrel following their stroke, we can see that their resting and ADP-induced P-selectin exposure is significantly lower by day-90 (S2C and S2D Fig). Furthermore, the significant reduction in P-selectin response to CRP-XL in the stroke patients seen at day-90 compared to day-0 (Fig 3C) could also be due to a general downregulation of P-selectin exposure or even shedding from the platelet surface after stroke [29].

At admission, GPVI-dimer expression was higher in all ischemic stroke subtypes and all hemorrhagic stroke patients (Fig 4). This is not surprising, as ischemic and hemorrhagic stroke patients share similar vascular risk-factor profiles (for example, hypertension and diabetes) and therefore would be expected to also demonstrate higher GPVI expression pre-stroke. Although there were only 15 hemorrhagic strokes, compared to 91 ischemic ones (excluding UD and Other), there were also no significant differences between total GPVI or GPVI-dimer expression between the ischemic or hemorrhagic strokes.

The stroke patients recruited in this study appear suitably representative of the broader population; however, one of the major limitations of this study is that they are older than the control population. We have corrected for this confounding factor by adjusting GPVI expression and P-selectin exposure data by age and MPV as described in the statistical methods section. Furthermore, we did a separate analysis of age and MPV unadjusted data, where stroke day-0 and control patients were pair-matched by age. This demonstrated in 62 age-matched [age: median (IQR) both groups, 67 (65–78)] stroke and control patients that GPVI-dimer expression (*P* = 0.003) as well as resting P-selectin exposure (*P* <0.0001) remain significantly higher in the stroke patients compared to controls (S2 Table). These results further consolidate that expression of the functional form of GPVI, the GPVI-dimer, is higher in patients who have had a stroke compared to healthy controls of the same age and that they have more activated circulating platelets.

The purpose of this study was to quantify GPVI-dimer expression on the platelet surface in stroke patients compared to healthy controls. We demonstrate that stroke patients overexpress GPVI-dimer at least until day 90 post-stroke, in all stroke sub-classifications, and that anti-thrombotic therapy, including antiplatelet drugs, anticoagulation or thrombolysis, do not affect GPVI expression. Further longitudinal work is needed to understand some of the associations illustrated in this study, especially regarding GPVI-dimer expression in the setting of vascular risk factors.

The ability of GPVI to interact with the two main ligands that drive thrombosis, collagen and fibrin, cements its role as a key platelet receptor in human thromboembolic disease. Our results intimate an important role for GPVI-dimer in thrombosis in both LAS and CES, and over-expression of GPVI-dimer in all ischemic stroke etiologies suggests that direct and selective inhibition of GPVI-dimer could be a promising therapy against ischemic stroke of different sub-types.

## Supporting information

S1 Fig The effect of thrombolysis on GPVI expression and P-selectin exposure. **A)** No significant differences were observed in GPVI expression between stroke patients who were thrombolyzed (n = 50) and those who were not (n = 79). **B)** Similarly, no significant differences were seen in GPVI expression if the blood was sampled pre- or post-thrombolysis. *P*-values were calculated using an unpaired t-test and the error bars represent the mean MFI of each of the cohorts ± SD. MFI = mean fluorescence intensity, ns = not significant. tPA = thrombolysis.
(JPG)

S2 Fig Comparison of the total GPVI, GPVI-dimer, and P-selectin exposure in day-0 (no anti-platelets) and the same individuals at day-90 who were discharged on clopidogrel. GPVI-dimer was significantly increased (**A**, *P* = 0.007) but total GPVI was not (**B**, *P* = 0.68). Both resting P-selectin (**C**, *P* = 0.001) and ADP-induced P-selectin were significantly lower at day-90 (**D**, *P* = 0.002), but CRP-XL–induced P-selectin was not (**E**, *P* = 0.90). Error bars represent median (Q_1_-Q_3_) %PP. *P-*values calculated using a paired t-test for parametric or Wilcoxon signed-rank test for non-parametric data. ns = not significant.
(JPG)

S1 Table Multiple regression analysis applying significant factors associated with GPVI-dimer expression in bivariate comparisons (Table 2 of manuscript). There was no multicollinearity between any of the tested variables in this model. A higher CHA_2_DS_2_-VASc score was significantly associated with GPVI-dimer expression (P = 0.03). Model summary: Adjusted R^2^ = 0.14, *P* = 0.001.
(DOCX)

S2 Table Each stroke patient was pair-matched to a control by age and GPVI expression and resting P-selectin exposure was calculated. This was done by choosing the first stroke and first control recruited for a specific age. If there was more than one stroke patient for a particular age, the next control recruited of the same age was included. Stroke patients without a corresponding age match were excluded. *P*-values were calculated using an unpaired t-test for parametric or Mann Whitney-U test for non-parametric data. GPVI-dimer expression (*P =* 0.003) and resting P-selectin exposure (*P* <0.0001) remain significantly raised between age-matched stroke and control participants.
(DOCX)
