# Supplementary figures and images for: Platelet surface receptor glycoprotein VI-dimer is overexpressed in stroke: The Glycoprotein VI in Stroke (GYPSIE) study results
Source: PLoS One. 2022 Jan 18;17(1):e0262695. doi: 10.1371/journal.pone.0262695 (PMC8765640; doi:10.1371/journal.pone.0262695)

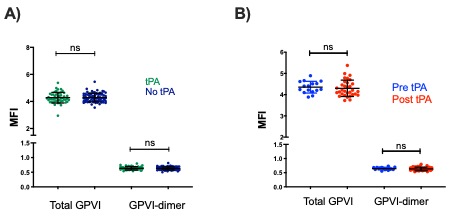

Supplement: S1 Fig — A) No significant differences were observed in GPVI expression between stroke patients who were thrombolyzed (n = 50) and those who were not (n = 79). B) Similarly, no significant differences were seen in GPVI expression if the blood was sampled pre- or post-thrombolysis. P-values were calculated using an unpaired t-test and the error bars represent the mean MFI of each of the cohorts ± SD. MFI = mean fluorescence intensity, ns = not significant. tPA = thrombolysis. (JPG) [file pone.0262695.s001.jpg]

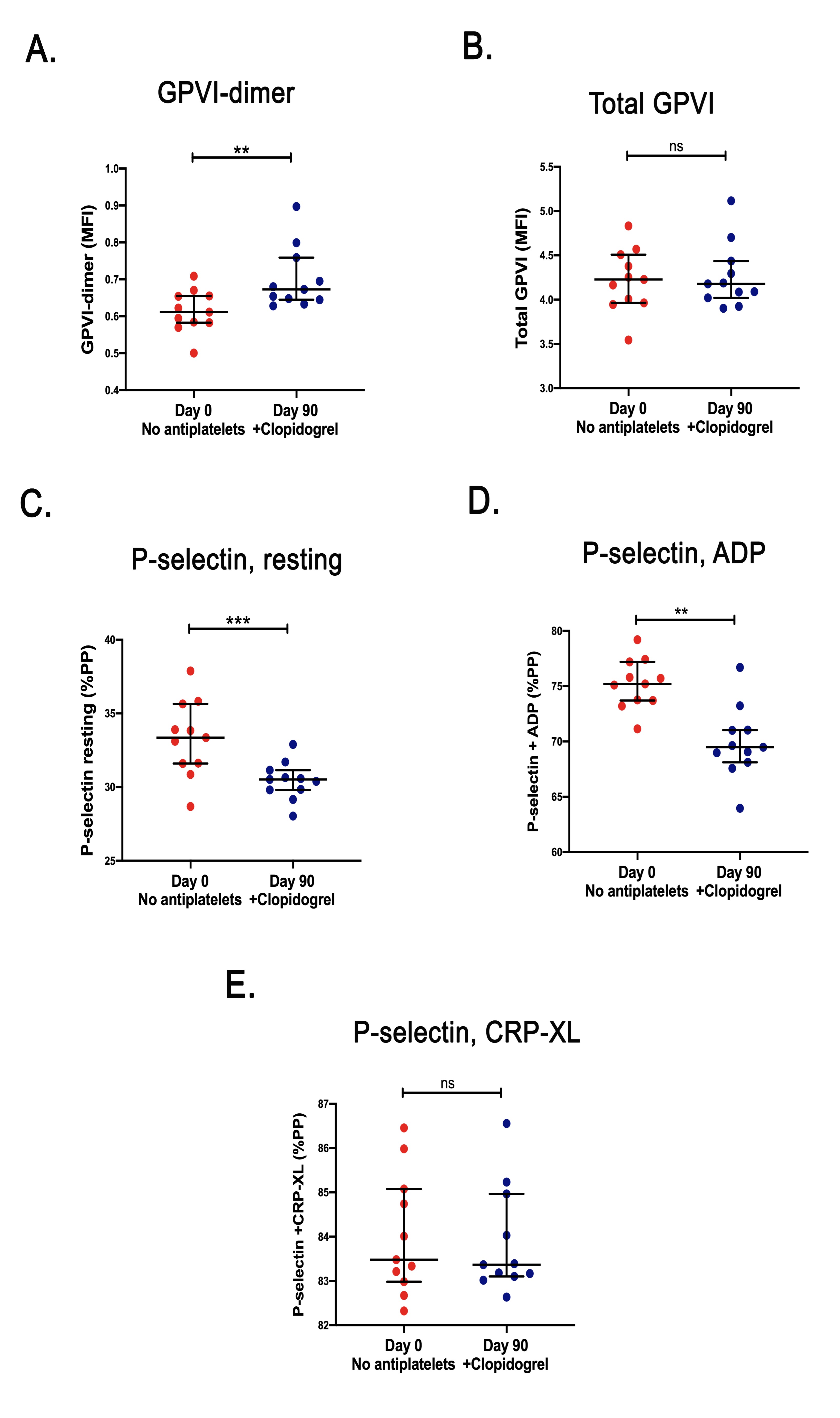

Supplement: S2 Fig — GPVI-dimer was significantly increased (A, P = 0.007) but total GPVI was not (B, P = 0.68). Both resting P-selectin (C, P = 0.001) and ADP-induced P-selectin were significantly lower at day-90 (D, P = 0.002), but CRP-XL–induced P-selectin was not (E, P = 0.90). Error bars represent median (Q1-Q3) %PP. P-values calculated using a paired t-test for parametric or Wilcoxon signed-rank test for non-parametric data. ns = not significant. (JPG) [file pone.0262695.s002.jpg]
