# Supplementary material for: Platelet surface receptor glycoprotein VI-dimer is overexpressed in stroke: The Glycoprotein VI in Stroke (GYPSIE) study results
Source: PLoS One. 2022 Jan 18;17(1):e0262695. doi: 10.1371/journal.pone.0262695 (PMC8765640; doi:10.1371/journal.pone.0262695)
Supplement: S1 Table — There was no multicollinearity between any of the tested variables in this model. A higher CHA2DS2-VASc score was significantly associated with GPVI-dimer expression (P = 0.03). Model summary: Adjusted R2 = 0.14, P = 0.001. (DOCX) [file pone.0262695.s003.docx]

S1 Table

| GPVI-dimer | **Coefficient B** | **Standard Error** | **Significance (*P*)** |
| --- | --- | --- | --- |
| **CHA_2_DS_2_-VASc score** | **0.008** | **0.004** | **0.03** |
| Atrial Fibrillation | 0.018 | 0.01 | 0.06 |
| Admission NIHSS score | 0.001 | 0.001 | 0.11 |
| Diabetes | 0.006 | 0.014 | 0.67 |
| Hypertension | 0.005 | 0.013 | 0.72 |
| Discharge mRS | -0.001 | 0.003 | 0.75 |
