# Supplementary material for: Platelet surface receptor glycoprotein VI-dimer is overexpressed in stroke: The Glycoprotein VI in Stroke (GYPSIE) study results
Source: PLoS One. 2022 Jan 18;17(1):e0262695. doi: 10.1371/journal.pone.0262695 (PMC8765640; doi:10.1371/journal.pone.0262695)
Supplement: S2 Table — This was done by choosing the first stroke and first control recruited for a specific age. If there was more than one stroke patient for a particular age, the next control recruited of the same age was included. Stroke patients without a corresponding age match were excluded. P-values were calculated using an unpaired t-test for parametric or Mann Whitney-U test for non-parametric data. GPVI-dimer expression (P = 0.003) and resting P-selectin exposure (P <0.0001) remain significantly raised between age-matched stroke and control participants. (DOCX) [file pone.0262695.s004.docx]

|  | **Control (n=62)** | **Stroke day-0 (n=62)** | **P** |
| --- | --- | --- | --- |
| Age (Q_1_-Q_3_) | 67 (65-78) | 67 (65-78) |  |
| Platelet GPVI expression (MFI)±SD | | | |
| Total | 4.23±0.70 | 4.23±0.69 | 0.97 |
| **Dimer** | 0.54± 0.15 | 0.62±0.14 | **0.003** |
| Median (Q_1_-Q_3_) P-Selectin Expression (%PP) | | | |
| **Resting** | 18.6 (15.7-20.7) | 29.6 (25.0-40.5) | **<0.0001** |

S2 Table
